# Subacute Combined Degeneration of the Spinal Cord Caused by an Impairment in the Functional Vitamin B12 Metabolic Pathway

**DOI:** 10.7759/cureus.73617

**Published:** 2024-11-13

**Authors:** Ahmed S Al-Jizani, Snehal Pathak, Punam Palit, Nkechi Achufusi

**Affiliations:** 1 Gastroenterology, Peterborough City Hospital, Peterborough, GBR; 2 Internal Medicine, Peterborough City Hospital, Peterborough, GBR; 3 Medicine, Peterborough City Hospital, Peterborough, GBR; 4 Neurology, Peterborough City Hospital, Peterborough, GBR

**Keywords:** adult neurology, all neurology, functional vitamin b12 deficiency, lower limb pain, sensory ataxia, spinal cord abnormalities, subacute combined degeneration, subacute combined spinal cord degeneration, vitamin b deficiency

## Abstract

Subacute combined degeneration (SCD) of the spinal cord is a neurological disorder marked by degeneration in the dorsal and lateral columns, commonly linked to vitamin B12 deficiency. This report presents a case of SCD caused by a functional vitamin B12 deficiency. Although the patient displayed symptoms such as ataxia, sensory deficits, and motor impairment, initial lab tests showed normal serum vitamin B12 levels, complicating the diagnostic process. A comprehensive assessment identified poor nutrition as the root cause affecting the body’s vitamin B12 metabolism. This case highlights the importance of considering SCD in patients with neurological symptoms, even when serum B12 levels appear normal. The report calls for greater awareness and thorough evaluation of vitamin B12 metabolic pathways in patients with unexplained neurological symptoms, emphasizing the need for clinicians to look beyond standard serum levels when assessing potential deficiencies.

## Introduction

Vitamin B12, also known as cobalamin, is a water-soluble vitamin that plays a crucial role in numerous physiological processes within the body [1]. The body requires vitamin B12 for proper metabolic function, including the conversion of carbohydrates, fats, and proteins into energy. Additionally, it supports the production of myelin, the protective sheath surrounding nerves, which is vital for efficient nerve signal transmission. A deficiency in vitamin B12 can lead to serious health issues, including anemia, neurological disorders, and cognitive decline [2].

Subacute combined degeneration (SCD) is a serious neurological disorder primarily associated with vitamin B12 deficiency. This condition results in progressive demyelination of the dorsal and lateral columns of the spinal cord, leading to significant motor and sensory impairments. Individuals with SCD often experience symptoms such as ataxia, sensory deficits, and weakness, which can severely impact mobility and quality of life. SCD is commonly observed in patients with pernicious anemia, malabsorption syndromes, or those following strict vegetarian or vegan diets, as these factors can lead to inadequate vitamin B12 levels [3]. Early diagnosis and intervention are crucial for preventing irreversible neurological damage, and making awareness of this condition is essential for healthcare professionals and patients. Understanding the underlying mechanisms and risk factors associated with vitamin B12 deficiency can aid in the timely recognition and management of SCD of the spinal cord [4].

This case report outlines a rare occurrence of SCD of the spinal cord in which consistently normal vitamin B12 levels delayed diagnosis, potentially leading to permanent neurological damage.

## Case presentation

Medical history and demographics

A 23-year-old woman presented with tingling sensations and pain in her feet, accompanied by unsteadiness and newly developed urinary incontinence. Her symptoms began eight months postpartum and progressively worsened. She experienced significant weight loss, decreasing from 90 kg during her pregnancy to 50.7 kg over 10 months. Her diet primarily consisted of takeaway foods, including pizza and burgers, along with large quantities of stimulant drinks such as Red Bull®, while her intake of vegetables and meat was minimal. She did not report any notable past medical history or the use of nitrous oxide. She also noted that she does not smoke, drink alcohol, or take any oral contraceptives.

A clinical examination, particularly focusing on the neurological assessment, revealed normal cranial nerve function but indicated sensory ataxia with decreased sensation extending to the ribcage. Additionally, there was impaired joint position sense in the lower limbs and mild weakness in the small muscles of her hands. Pain sensation was diminished in the left leg, and reflexes were challenging to elicit, though they were subtly present. Assessment of the plantar reflexes was not possible due to pain in her feet.

Investigations

Blood tests performed during the initial evaluation showed normal serum vitamin B12 levels at 342 ng/L and folate levels at 3.5 µg/L. Additionally, all other hematological and biochemical parameters were satisfactory and within the normal range (Table 1). Given the normal results, a preliminary diagnosis of Guillain-Barré syndrome and transverse myelitis was considered. The patient was offered a lumbar puncture for further evaluation, but she declined the procedure. Consequently, she was scheduled for an outpatient follow-up with the neurology team and an urgent MRI of the spine.

**Table 1:** Patient’s vitamin/mineral levels and full blood test. All blood tests, including vitamin B12 and folate levels, were within the normal range at her initial presentation to the hospital. ALP, alkaline phosphatase; eGFR, estimated glomerular filtration rate

Test	Result	Reference range
Vitamin B12	342	200-771 ng/L
Folate serum	3.5	>3.0 µg/L
Albumin	43	35-50 g/L
ALP	92	30-130 IU/L
Calcium	2.27	2.20-2.60 mmol/L
Adjusted calcium	2.31	2.20-2.60 mmol/L
Phosphate	1.39	0.80-1.50 mmol/L
Magnesium	0.79	0.75-1.05 mmol/L
White cell count	13.8	4.0-11.0 x 10^9^/L
Hemoglobin	141	115-165 g/L
Platelets	425	150-400 x 10^9^/L
RBCs	4.70	3.8-5.8 x 10^12^/L
Hematocrit	0.412	0.360-0.460 L/L
Mean corpuscular volume	87.7	80-100 fL
Mean corpuscular hemoglobin	30.0	27-33 pg
RBC distribution width	14.8	11.5-15.0%
Mean platelet volume	10.4	9.0-12.1 fL
Immature granulocytes	0.3	0-1%
Neutrophils	7.9	1.8-7.7 x 10^9^/L
Lymphocytes	4.7	1.4-4.8 x 10^9^/L
Monocytes	0.8	0.1-0.8 x 10^9^/L
Eosinophils	0.4	0.1-0.6 x 10^9^/L
Basophils	0	0-0.1 x 10^9^/L
Sodium	136	133-146 mmol/L
Potassium	4.2	3.5-5.3 mmol/L
Chloride	106	95-108 mmol/L
Creatinine	66	45-84 µmol/L
eGFR	90	>60 mL/min/1.73m²
Urea	4.1	2.5-7.8 mmol/L

**Figure 1:**
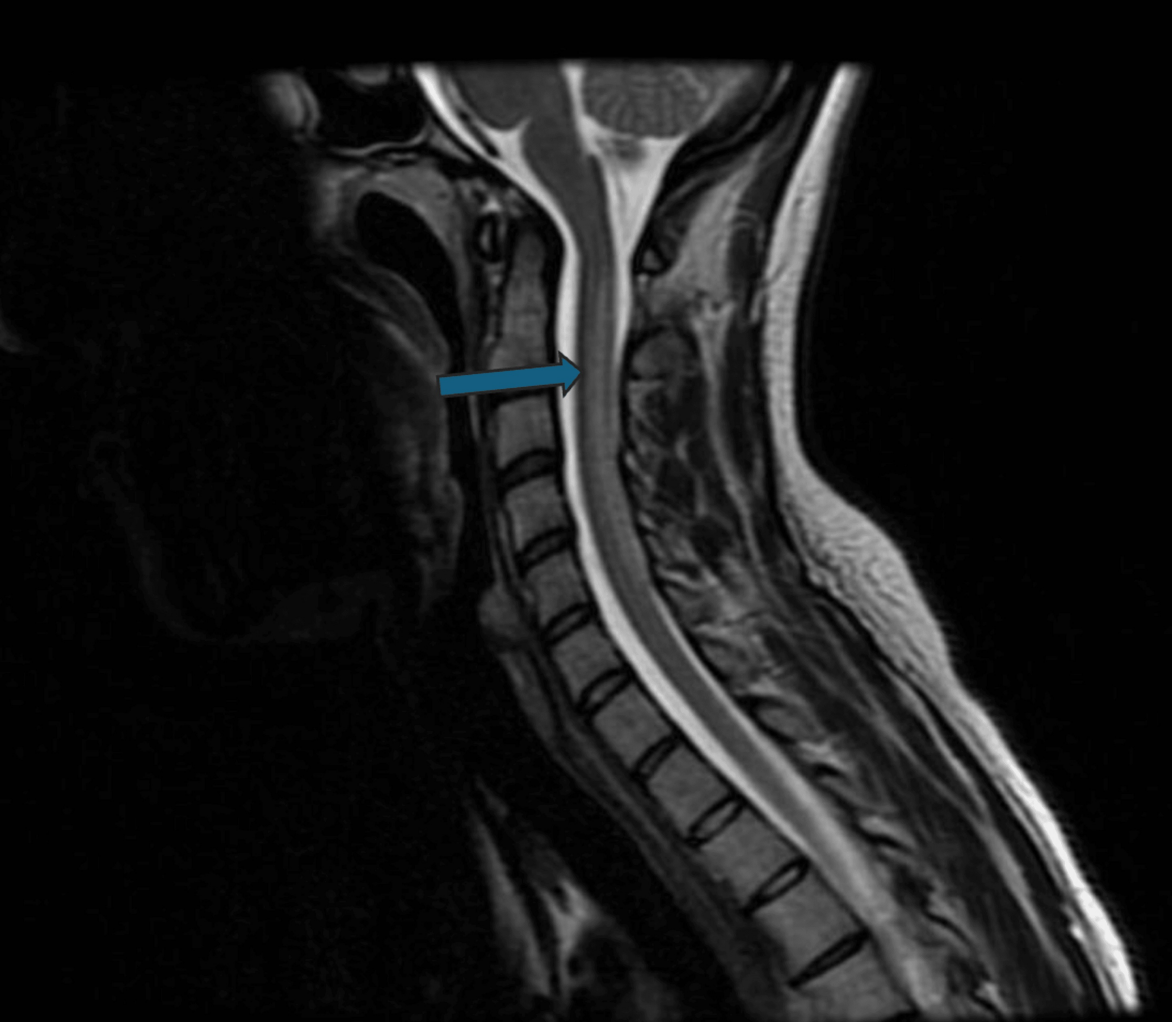
MRI of the cervical spinal cord showing demyelination. This T2-weighted MRI scan shows hyperintensity in the dorsal aspect of the cervical spinal cord. The hyperintensity is most prominent at the C1 to C5 levels, which suggests demyelination or inflammation in this region (blue arrow).

**Figure 2:**
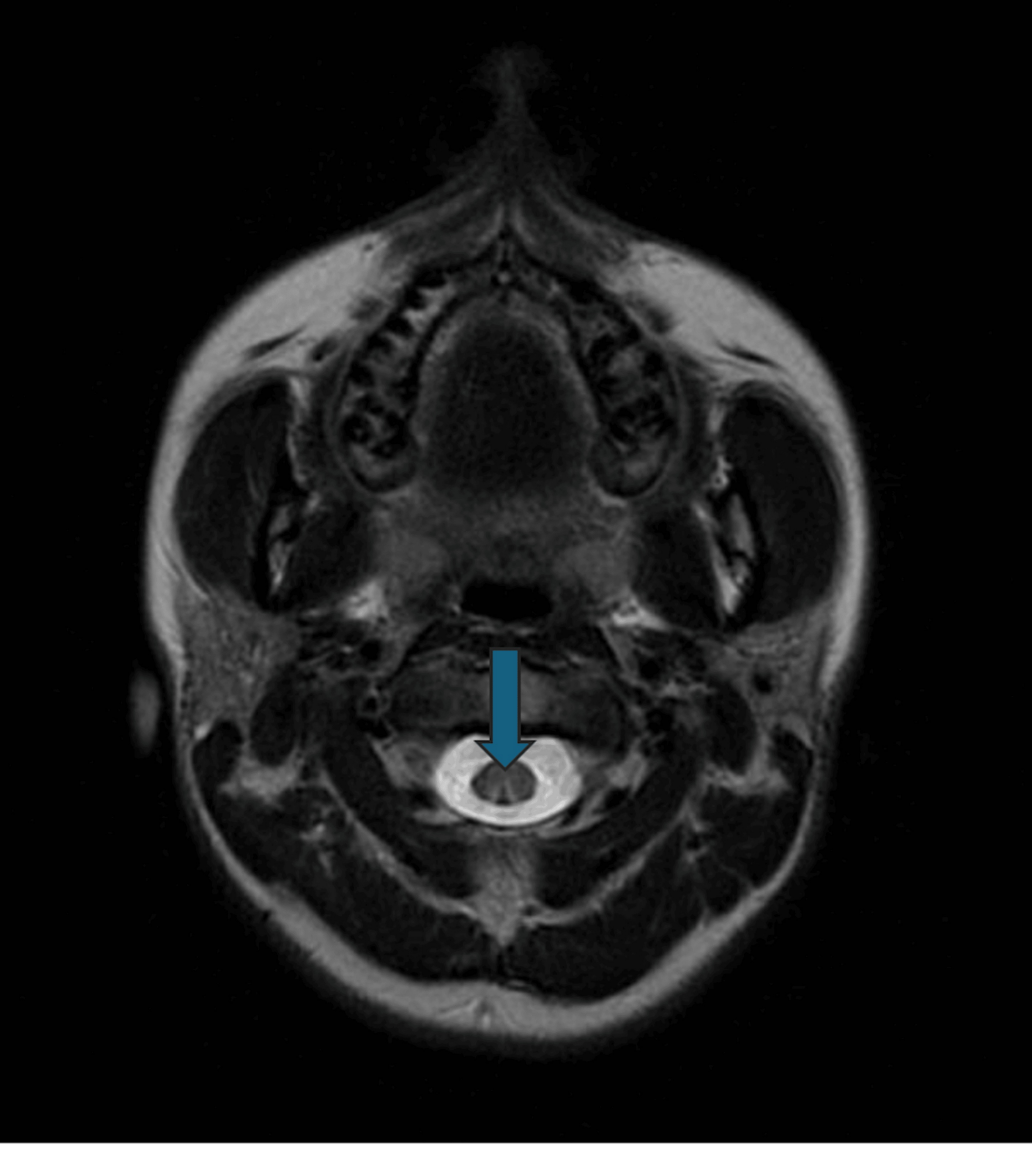
MRI of the spinal cord showing "inverted V sign." The axial T2 view of the spinal cord displays the classic "inverted V sign." This finding is characteristic of SCD, where the demyelination affects the dorsal columns symmetrically, creating this distinctive V-shaped pattern on axial imaging (blue arrow). SCD, subacute combined degeneration

Following the outpatient MRI of the spine, results revealed hyperintensities in the dorsal columns of the cervical and thoracic spine, along with an "inverted V" sign on the axial view. These imaging findings increased the suspicion of SCD (Figures 1, 2).

The patient was scheduled for a repeat blood investigation approximately 10 days after the initial investigations. These tests confirmed elevated homocysteine levels of 38.3 µmol/L, while vitamin B12 and folate levels remained normal indicating a disruption in the B12 metabolic pathway. Other vitamin tests, including vitamin A, vitamin E, and copper levels, were within normal ranges. Additionally, antibodies for syphilis, aquaporin-4, and myelin oligodendrocyte glycoprotein tested negative (Table 2). The methylmalonic acid (MMA) level was not measured.

**Table 2:** Repeated blood investigations, including vitamin B12 levels. Blood test showing high total homocysteine levels and normal vitamin B12 and folate levels. ANA, anti-nuclear antibody

Test	Result	Reference range
Vitamin B12	342	200-771 ng/L
Folate	3.5	>3.0 µg/L
Total homocysteine	38.3	0-16.0 µmol/L
Vitamin A	1.64	0.99-3.35 µmol/L
Vitamin E	20.0	9.5-41.5 µmol/L
ANA	0.1	<0.7
Serum copper	16.8	11.0-25.0 µmol/L

Treatment

The patient's clinical presentation, along with the MRI findings and elevated homocysteine levels, supported the diagnosis of SCD of the spinal cord due to a disrupted B12 pathway and elevated homocysteine levels. These factors collectively pointed to this condition as the underlying cause of her neurological deficits. After the diagnosis, she was promptly initiated on a complete course of vitamin B12 injections and immediately referred to the neurophysiotherapy team. She was also started on gabapentin for pain management.

Outcome and follow-up

During her follow-up after approximately four months, the patient exhibited noticeable signs of improvement, particularly in sensory function. Vibration sense, which had previously been undetectable, was now felt at the right ankle, although it remained weak on the left side. Pinprick sensation was also evaluated and appeared nearly normal, indicating a positive trend in sensory recovery. She also had a follow-up blood test that revealed normal homocysteine levels after treatment (Table 3).

**Table 3:** Follow-up blood tests after treatment. Blood investigations, including the homocysteine level, were repeated after the treatment, showing homocysteine within the normal range.

Test	Result	Reference range
Vitamin B12	500-700	200-771 ng/L
Folate	4.0-6.0	>3.0 µg/L
Total homocysteine	10-15	0-16.0 µmol/L
Methylmalonic acid	0.35	0.08-0.56 µmol/L

## Discussion

This case report discusses a young patient with a history of prolonged poor diet who developed sudden neurological symptoms following pregnancy and significant weight loss. Imaging studies, high homocysteine levels in the blood tests coupled with inadequate nutrition confirmed SCD of the spinal cord due to functional B12 deficiency. 

Vitamin B12 deficiency can result from various factors affecting absorption, metabolism, or intake. Strict vegetarian or vegan diets, malabsorption disorders such as celiac disease, and pernicious anemia, which impairs intrinsic factor production, are common causes. Additionally, surgical procedures, age-related factors in older adults, certain medications, chronic alcoholism, and rare genetic disorders can contribute to deficiency. Understanding these factors is essential for accurately diagnosing and effectively managing vitamin B12 deficiency [1]. Not everyone with a vitamin B12 deficiency will develop SCD. Only around 14.8% of individuals with vitamin B12 deficiency tend to experience SCD of the spinal cord [4].

Cobalamin is crucial for various metabolic pathways in the body, primarily occurring in the liver, which stores and processes this vitamin. Cobalamin undergoes a complex process, starting with absorption in the small intestine, followed by transport via the protein transcobalamin II (TCII), and concluding with conversion into two active coenzymes: methylcobalamin and adenosylcobalamin [5]. Methylcobalamin acts as a cofactor for methionine synthase, facilitating the conversion of homocysteine to methionine. This pathway is essential for DNA synthesis and methylation processes [6]. Adenosylcobalamin functions as a cofactor for methylmalonyl-coenzyme A (CoA) mutase, which converts methylmalonyl-CoA to succinyl-CoA, a critical step in the metabolism of certain amino acids and fatty acids. This pathway is important for energy production and the proper functioning of the nervous system [6].

Functional vitamin B12 deficiency occurs when the body does not have enough vitamin B12 to meet its needs, despite normal serum levels. Serum B12 levels are not reliable markers for physiological stores. Most B12 measurement assays only measure the protein-bound form of B12, which is unavailable to tissues. A deficiency is possible even with borderline or normal serum B12 levels, so measuring metabolite levels is required. A meta-analysis showed that one-third of patients with SCD have normal or high serum B12 levels [3]. Elevated levels of MMA can indicate a functional deficiency, as MMA accumulates when vitamin B12 is not available for metabolism. Likewise, increased homocysteine levels may suggest a functional deficiency because vitamin B12 is essential for converting homocysteine into methionine [7].

Vitamin B12 is vital for the synthesis of myelin, the protective covering that encases nerve fibers. Myelin, made up of lipids and proteins, is essential for effective nerve signal transmission. Adequate myelination allows electrical impulses to travel smoothly along nerve fibers, as myelin serves as an insulator that enhances the speed of nerve signal conduction. A deficiency in vitamin B12 can disrupt myelin production, resulting in neurological deficits [8].

Another condition characterized by elevated homocysteine levels is TCII deficiency, a rare inherited disorder that impairs vitamin B12 (cobalamin) transport within the body. TCII is an essential protein that binds to vitamin B12, facilitating its movement from the bloodstream into cells, where it is critical for DNA synthesis and RBC production. Without sufficient TCII, vitamin B12 cannot effectively reach the cells, resulting in deficiencies even when dietary intake is adequate [9].

Some other differentials to consider in this similar clinical presentation include infectious causes such as HIV-1 associated myelopathy and neurosyphilis, autoimmune causes such as autoimmune myelopathy, Guillain-Barre syndrome, and demyelinating causes such as multiple sclerosis, neuromyelitis optica [10].

SCD exhibits characteristic MRI findings in the spine, including hyperintense regions in the dorsal and lateral columns of the cervical and thoracic spinal cord. These hyperintensities suggest demyelination and edema. Additionally, the "inverted V sign," a classic radiological feature observed in axial T2-weighted images, may be present, along with the “pair of binoculars sign” and the “dot sign” [11].

SCD is a progressive condition, so making a timely diagnosis is essential. Without prompt intervention and active treatment, the disease may advance, resulting in irreversible neurological damage. Treatment involves vitamin B12 injections, which can usually halt disease progression and may even allow for partial recovery of neurological function if started early [12]. 

## Conclusions

This case highlights the necessity of considering functional vitamin B12 deficiency in patients presenting with neurological symptoms, even when serum B12 levels appear normal. Persistent normal B12 levels accompanied by ongoing neurological symptoms should prompt further investigation into homocysteine and MMA levels to assess vitamin B12 metabolism. Early identification of these markers is crucial to prevent irreversible neurological damage associated with SCD of the spinal cord. Timely interventions, such as vitamin B12 supplementation, can halt disease progression and potentially allow for partial recovery of neurological function. This case highlights the importance of comprehensive assessments to identify vitamin B12-related issues in patients with unexplained neurological deficits.
